# Microneedling and Topical Retinyl Palmitate for Acne Scars: A Preliminary Split-Face Study with Placebo Control

**DOI:** 10.3390/jcm15062185

**Published:** 2026-03-13

**Authors:** Aleksandra Tobiasz, Alina Jankowska-Konsur, Danuta Nowicka

**Affiliations:** University Centre of General Dermatology and Oncodermatology, Wroclaw Medical University, 50-556 Wroclaw, Poland

**Keywords:** acne scars, microneedling, retinyl palmitate, split-face study

## Abstract

**Background**: Acne scars remain a very common complaint in dermatology practices. Even though many treatment options are available, proper treatment remains a challenge. Complex treatment methods that are based on the synergy effect are the ones that result in better effects and patient satisfaction. **Methods**: Three healthy female patients with a total of 106 atrophic acne scars were recruited to the split-face study with placebo control, where a series of three microneedling procedures in monthly intervals combined with 5% retinyl palmitate-loaded oleogel was compared to the same microneedling protocol with placebo. Patients’ quality of life was measured using the Dermatology Life Quality Index (DLQI) and Skindex-29 questionnaires. Patients’ satisfaction with treatment and intensity of post-procedure symptoms were assessed as well. **Results**: In clinical evaluation, a modest effect was observed regarding the reduction in atrophic acne scars, whereas moderate-to-marked improvement in acne scar reduction was noted by the patients. Additionally, mild to marked improvement was noted by patients regarding skin quality, moisture level, elasticity, and skin tone. No significant side effects were noted. All the above resulted in good patient satisfaction with the treatment, and willingness to repeat the procedures again. No significant differences regarding acne scar reduction, treatment-related symptoms, and skin quality improvement were noted between active substance and placebo-treated sides of the face. **Conclusions**: Microneedling remains a key method in the therapeutic arsenal for acne scarring. By combining it with 5% retinyl palmitate-loaded oleogel modest effects can be noted after a series of three procedures, with good overall treatment tolerability and patients’ satisfaction.

## 1. Introduction

As a very high prevalence of acne vulgaris remains an actual issue, acne scarring represents a common therapeutic challenge [1,2,3]. What makes the search for an effective acne scar treatment such a major concern is the psychological burden on patients who struggle with it [4,5,6].

Lesions in acne vulgaris can result in atrophic and hypertrophic scars. The more common ones are atrophic ones. Depending on their morphology, they can be divided into three types: boxcar, icepick, and rolling scars. Mechanisms behind acne scarring differ. In atrophic scarring, lesion formation occurs due to prolonged inflammation, which correlates with increased secretion of numerous inflammatory factors that are destructive to the structures of sebaceous glands and cause collagen degradation. On the other hand, in hypertrophic scars, because of inflammation, a disbalance in the inflammatory response occurs, which results in overexpression of transforming growth factor-beta 1. Its excess leads to an uncontrolled fibroblast response [7].

Taking into consideration the type of scar and pathophysiology behind its formation, many treatment options have been studied to date. Options are very wide-ranging, from mechanical subcision, microneedling, through application of various chemical peels of different depth, injecting autologous formulations like platelet-rich plasma, nano fat, and injectables (fillers, stimulators) to stimulate collagen remodeling and for volume restoration. Finally, modern technology devices such as lasers and radiofrequency microneedling are used [8,9,10]. What is important within laser technology is that various types of lasers can be distinguished—ablative, nonablative, vascular. Such a variety of mechanisms of action enables the treatment of various type of scars [11]. All these modalities can be used in monotherapy and combined. What has been observed is the fact that combining various treatment options gives superior results. Microneedling has already been present in the dermatology treatment world for a few decades. Microneedling uses the potential of skin to regenerate and remodel after a mechanical stimulus, which creates microchannels. Of course, depending on the tool being used, such channels can have a different depth, number, and width. What is important is that such channels can also mediate various active substances into deeper layers of the skin. Nowadays, many automatic devices for this procedure exist, with the option of adjusting proper parameters. It gives better controlled results and less trauma [12,13]. One of such devices, widely used in dermatology and aesthetic medicine, is Dermapen 4^®^. This device uses disposable cartridges with 16 needles, offers a possibility to adjust the depth of the procedure up to 3 mm, and to select various speed modes in which the needles operate [14]. Even though the positive effects of microneedling in the monotherapy of acne scars have been reported [15], combining it with a formulation that would potentiate such an effect seems to be a superior solution [16]. Vitamin A and its derivatives are used in a wide variety of dermatological conditions [17,18]. Their skin remodeling potential in acne scarring has been described [19]. Combining retinyl palmitate with microneedling seems to be a very promising approach to acne scar treatment. To the best of the authors’ knowledge, no similar study could be found.

The aim of the study was to assess and compare the effects, tolerability, and safety of microneedling in combination with 5% retinyl palmitate-loaded oleogel on one side of the face vs. placebo on another side of the face in patients with atrophic acne scars.

## 2. Materials and Methods

The study was approved by the Ethics Committee of Wroclaw Medical University (No. KB 592/21) and performed in accordance with the Declaration of Helsinki. The study assumed recruiting healthy individuals with atrophic acne scars. Exclusion criteria were acne scar therapy, active infectious disease, tendency to hypertrophic/keloid scars, pregnancy and lactation, major health issues (active oncological disease, oncological treatment, unregulated diabetes), allergy to any of the substances used during treatment, and ongoing acne treatment.

After explaining all the details of the study to the patients, informed, handwritten consent was obtained. The experiment protocol included basic physical examination and dermatological assessment of skin lesions during each visit. The number and type of scars were examined, their severity was evaluated using Goodman and Baron’s qualitative and quantitative scale [20,21]. The mentioned scales have a global character, assessing the severity of scarring on the whole face. The severity of scarring on each side of the face was assessed clinically.

The study consisted of 3 microneedling sessions combined with 5% retinyl palmitate-loaded oleogel on the right side of the face and placebo (pure oleogel) on the left side of the face. Formulations were manufactured in cooperation with the Department of Drug Form Technology, Wroclaw Medical University. Utilized ingredients were of pharmacopeial grade—retinyl palmitate oleic solution was purchased from Caesar & Loretz GmbH—Caelo (Hilden, Germany), Oleogel from Actifarm (Warsaw, Poland). A detailed study describing the manufacturing and parameters of formulations will be described in a subsequent publication.

Procedures were performed in monthly intervals with a follow-up visit one month after the last treatment. Before the procedure, the face was properly cleansed using a delicate syndet and disinfected. Each treatment session was performed, focusing on atrophic scars, using Dermapen 4^®^ (DermapenWorld, Sydney, Australia), on depth of 1.0–1.5 mm, 3–4 passes, with a maximal frequency until pinpoint bleeding. Formulations were removed from the skin surface right after the microneedling session. Patients were instructed to use gentle syndets, neutral moisturizers and photoprotection during the study. Patients’ quality of life was also measured, before the first procedure and during a follow-up visit using Dermatology Life Quality Index (DLQI) [22,23] and Skindex-29 questionnaires [24,25]. Patients’ satisfaction and final overall impressions (treatment effects, post-treatment symptoms intensity) after a series of procedures were evaluated considering each side of the face and the overall assessment.

## 3. Results

Three healthy female patients, phototype 2, with a history of severe acne vulgaris in the past were recruited to the study. All patients had numerous atrophic acne scars of various types, mostly on both cheeks; in one of the patients, scars were also present on the temples and jawline. Patients’ age as well as Goodman and Baron’s qualitative and quantitative scale scores before and a month after a series of three procedures, are presented in Table 1. In the clinical assessment, a modest reduction in acne scars was observed, manifested mainly by a slight shallowing of existing scars and improvement in skin texture.

No significant difference in scar reduction between the active substance and placebo side of the face was observed.

**Figure 1:**
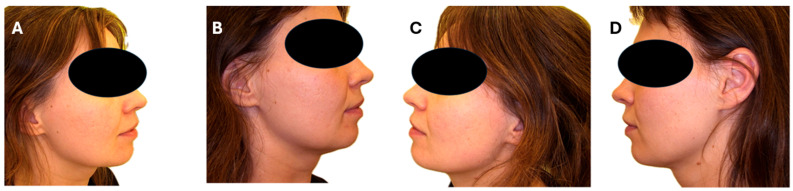
Visual presentation of treatment results: (**A**), right side of the face before treatment; (**B**), right side of the face 1 month after a series of three procedures; (**C**), left side of the face before treatment; (**D**), left side of the face 1 month after a series of three procedures.

A small, one-point reduction in Goodman and Baron’s quantitative scale was noted after a series of procedures in patients II and III; no change in score was noted in patient number I. Figure 1 presents photographs of the patients before and after treatment. The type and number of scars before and after the series of procedures are summarized in Table 2. The number of acne scars before treatment was 106 and after was 96. A reduction in macular lesions in patient III and rolling scars in patients I, II, and III was observed.

Table 3 summarizes changes in the patients’ quality of life before and after the series of procedures, as well as post-procedure recovery, satisfaction with treatment and willingness to repeat the treatment. Results demonstrate different levels of quality-of-life improvement, willingness of patients to repeat the treatment, adequate to rather short recovery time assessment and all patients being rather satisfied with the treatment.

Post-procedure recovery assessment: 1—definitely too long, 2—rather long, 3—appropriate, 4—rather short, 5—very short.

Satisfaction level: 1—completely dissatisfied, 2—rather dissatisfied, 3—neutral, 4—rather satisfied, 5—very satisfied.

**Figure 2:**
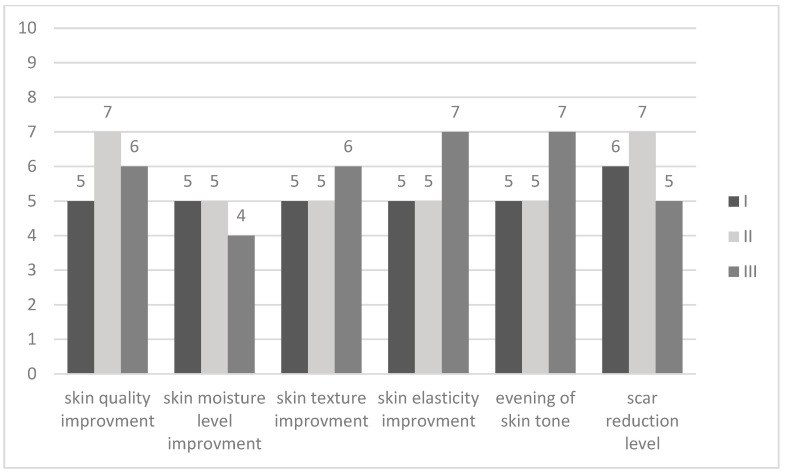
Patients’ overall assessment of effects after a series of three procedures. 0 no improvement, 1–2 barely perceptible improvement, 3–4 mild improvement, 5–6 moderate improvement, 7–8 marked improvement, 9 almost complete improvement, 10 complete improvement.

Patients were asked to assess certain parameters such as improvement of skin quality, moisture level, skin texture, elasticity, skin tone, and scar reduction level on a 0–10 scale for each facial side (right and left) and overall. As identical values were obtained for the left side, right side, and overall assessment, Figure 2 presents a single bar.

Patients have stated moderate-to-marked improvement in scar reduction, moderate-to-marked improvement in skin quality, texture, elasticity, and skin tone, and mild-to-moderate improvement in skin moisture level.

**Figure 3:**
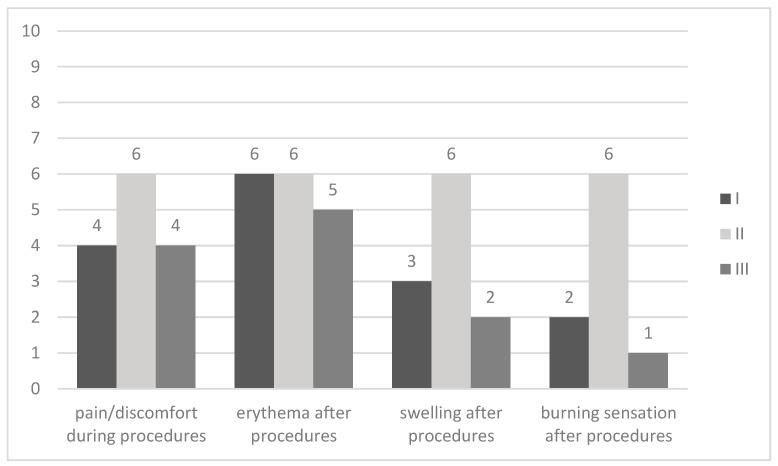
Patients’ overall assessment of procedure-related symptoms. 0 absent, 1–2 minimal, 3–4 mild, 5–6 moderate, 7–8 severe, 9 very severe, 10 unbearable.

Figure 3 summarizes patients’ assessment of procedure-related symptoms. Similarly, like before, certain parameters were measured separately for each facial side and overall. Like in the case of previous parameters, patients graded each parameter with the same value for each facial side, and overall assessment; therefore, Figure 3 uses one bar. Patients stated mild-to-moderate pain/discomfort during procedures, moderate erythema after procedures, minimal-to-moderate swelling after procedures, and minimal-to-moderate burning sensation after procedures. All symptoms were temporary and characteristic of such a procedure. No side effects were reported by the patients.

## 4. Discussion

Even though numerous studies have been published to date regarding different acne scar treatment options, an optimal, effective protocol still has not been established.

One of the reasons is the complex character of acne scars and their varying severity, which requires a multimodal, tailored approach. Another issue is the different potential of patients to develop acne scars, and therefore, it can be assumed that there is potential to react to certain treatment options as well. The study by Holland et al. researched patients susceptible to acne scarring, identifying that in this group of patients, prolonged inflammation in resolving lesions is a significant scar formation-stimulating factor [26]. Different types of acne scars also have varying potential to respond to certain treatment options [27]. Observed reduction in macular and rolling scars is consistent with previously published studies. For example, in the study of Rana et al., the efficacy of microneedling with 70% glycolic acid was compared to microneedling alone and rolling scars showed maximal improvement [28]. As microneedling can cause hyperpigmentation reduction, macular scars can also appear to be much less apparent, like in the study of Wan et al. where promising results regarding a reduction in post-acne hyperpigmentation was observed in patients who underwent microneedling combined with Panax ginseng-derived exosomes [29].

Combining microneedling with various active substances has already become a standard procedure. In acne scarring, numerous publications assessing the effectives of microneedling combined mainly with platelet-rich plasma (PRP) [30,31,32,33] and chemical peels [28,34,35,36] can be found, indicating the superiority of such a combination vs. microneedling in monotherapy.

To the authors’ best knowledge, there is no similar study published to date researching the effect of microneedling in combination with retinyl palmitate or any kind of retinoid in general in acne scars. Single publications exist, like the one by Bergmann et al. [37], where the effectiveness of combining 5% retinoic acid solution with microneedling vs. 5% retinoic acid solution alone was examined in the treatment of melasma. Topical tretinoin had also been used as a part of more complex acne scar treatment protocols in a limited number of publications. Like the one by Zonunsanga et al., where the study focused on microneedling combined with 35% glycolic acid, accompanied by everyday use of 0.05% tretinoin with 12% glycolic acid [38]. On the other hand, in a study by Garg et al., 0.05% tretinoin was used as part of the everyday routine and right after the dermarolling procedure in a protocol which also included 15% trichloroacetic acid (TCA) peel and subcision [39].

In the present study, microneedling was of our great interest because of its minimal downtime and good tolerability. Interestingly, a systematic review by Nobari et al. analyzing various needling methods (RF-needling, meso-needling, microneedling) and ablative fractional lasers (CO_2_ and erbium YAG) in the treatment of atrophic and hypertrophic scars showed no statistically significant difference between the two methods in 60% of analyzed studies, and both methods were effective [40].

The novel character of the study resulted in focusing on the assessment of tolerability, safety, and efficacy of such a protocol. No difference in clinical assessment by the physician and in patients’ perspective was observed regarding tolerability between active substance and the placebo site. It is a positive observation regarding possible side effects that can be caused by combining vitamin A and its derivatives with invasive procedures.

On the other hand, no superior effect in clinical evaluation was noticed regarding retinyl palmitate and placebo sites in terms of acne scar reduction. Chosen retinyl palmitate is a very gentle ester, which must undergo a few chemical changes until it is transformed into an active metabolite. Even though having a very gentle profile of action, it still exceeds some level of activity on fibroblasts [41]. Acne scars of various types were assessed in the study. Observed clinical improvement was modest and mainly regarded reduction in shallow scars and skin tone evening. In contrast, patients’ perception of scar reduction was much greater than the clinical evaluation.

A study by Hayashi et al. investigated the effect of acne scars on patients’ quality of life and it stated that so-called “mini-scars” are just as bothersome for patients as larger scars [5]. We can assume that, similarly, even minimal changes in acne scar appearance can be significant to a patient.

Ultimately, in the patients participating in the current study, even a small reduction in scar appearance had a significant impact on patients’ satisfaction level and perception of scar reduction. Furthermore, no significant score reduction evaluated by Goodman and Baron’s qualitative and quantitative scales was noticed.

Acne scar assessment scales, even ones considered as a standard in acne scar assessment, such as the ones used in this study, have certain limitations. In each group of patients with mild, moderate, or severe scarring there are patients closer to the lower and higher score range limits. In such cases, small changes that are still noticeable, even significantly by the patient, can have no representation in the scale score. Such scales cannot reflect subtle skin texture improvement as well. Certain limitations of acne grading scales have also been already described in the literature, taking into account that it is an area that still requires development [42].

In the current study, patients were subjected to three procedures, which is an optimal number for observing the first results of microneedling. A study by Alster et al. examined microneedling in scar treatment. One to six procedures were performed, resulting in stating that 2.5 treatments was an average number to notice a significant effect [43]. As it was stated before, in the current study, clinically assessed effects after a series of three procedures were noticeable and modest; it can be assumed that by performing more procedures, even greater effectiveness could be achieved.

The present study not only included clinical aspects, but also psychological aspects and those connected to patient satisfaction with treatment, finding a tendency towards lower scores in quality-of-life questionnaires after a series of procedures, which indicates improvement in quality of life and good satisfaction with the procedures.

The approach of the presented study provides a complete, holistic overview, which is of great importance considering what a burden acne scars can be to the patients and how minimally invasive procedures with relatively short downtime and good tolerability are preferred by patients [4,6,44].

The study has its limitations. The first one is a small number of patients. The study assumed a completely novel combination of retinyl palmitate-loaded oleogel and microneedling, which has not been described so far in patients with acne scars. Moreover, microneedling is an invasive procedure, which must be performed with caution in combination with novel formulations. The given reasons require the choice of preliminary study protocol. Consequently, a study focusing on the evaluation of safety and tolerability of such a procedure was of major priority and a small number of patients was selected. Another limitation was a relatively short follow-up (one month)—of course, to assess full scar remodeling potential, a longer follow-up period should be considered. Still shorter, a one month follow-up is sufficient in terms of safety and tolerability examination, as well as early clinical response evaluation. Certainly, final conclusions about differences in effectiveness between placebo and active substance sites could be made after longer follow-up observation. Considering the relatively homogeneous character of studied cohort—all female patients of the same phototype, and in similar age group—the obtained results could be extrapolated only in patients of similar characteristics.

Taken together, further studies should include a larger group of patients, with wider demographic data, including various skin phototypes, and including a longer follow-up time.

## 5. Conclusions

Retinyl palmitate-loaded oleogel combined with microneedling in a series of three procedures exceeds good tolerability and modest clinical efficacy. Microneedling procedures series with active substance/placebo provided a decent effect in patients’ perception, with a good satisfaction level and efficacy, which resulted in a better quality of life for the patient. This shows how even small changes can be significant to a patient and how mild aggressiveness of treatment makes it preferable.

## Figures and Tables

**Table 1:** Scar severity assessment before and after a series of procedures.

Patient	Age	Goodman and Baron’s Qualitive Scale Before Treatment	Goodman and Baron’s Qualitative Scale After Treatment	Goodman and Baron’s Quantitative Scale Before Treatment	Goodman and Baron’s Quantitative Scale After Treatment
I	29	2–3	2–3	11	11
II	34	3	3	12	12
III	35	2–3	2–3	12	11

**Table 2:** Scar number assessment before and after a series of procedures.

Patient	Macular Scars		Rolling Scars		Boxcar Scars		Icepick Scars	
	Before	After	Before	After	Before	After	Before	After
I	0	0	17	15	12	12	1	1
II	5	7	16	13	18	18	5	5
III	5	0	10	8	15	15	2	2

**Table 3:** Quality-of-life evaluation before and after series of procedures.

Patient	DLQI Before	DLQI After	Skindex-29 Before	Skindex-29 After	Post-Procedure Recovery Assessment	Satisfaction Level After Series of Procedures	Willingness to Repeat Procedures
I	3	1	64	46	3	4	positive
II	3	0	87	80	3	4	positive
III	2	2	49	44	4	4	positive

## Data Availability

The data supporting the findings of this study are not publicly available due to ethical, legal, and privacy restrictions.

## Abbreviations

The following abbreviations are used in this manuscript:

DLQI	Dermatology Life Quality Index
PRP	Platelet-rich plasma
TCA	Trichloroacetic acid
